# Transcriptional Response to Fasting Studied in the Liver of Mice That Express Phosphorylation Resistant Perilipin 5

**DOI:** 10.1210/endocr/bqaf075

**Published:** 2025-04-16

**Authors:** Corinne E Bovee, Ryan P Grandgenett, Michelle B Trevino, Sucharita Dutta, Spencer J Peachee, Shayla Kopriva, Farakh Haider, Siming Liu, Gourav Bhardwaj, Christie Penniman, Brian T O’Neill, Yumi Imai

**Affiliations:** Department of Internal Medicine, Carver College of Medicine, University of Iowa, Iowa City, IA 52242, USA; Fraternal Order of Eagles Diabetes Research Center, University of Iowa, Iowa City, IA 52242, USA; Endocrinology Section, Iowa City Veterans Affairs Medical Center, Iowa City, IA 52246, USA; Department of Internal Medicine, Carver College of Medicine, University of Iowa, Iowa City, IA 52242, USA; Fraternal Order of Eagles Diabetes Research Center, University of Iowa, Iowa City, IA 52242, USA; Endocrinology Section, Iowa City Veterans Affairs Medical Center, Iowa City, IA 52246, USA; Department of Internal Medicine, Eastern Virginia Medical School, Norfolk, VA 23507, USA; Leroy T. Canoles Cancer Research Center, Eastern Virginia Medical School, Norfolk, VA 23507, USA; Department of Internal Medicine, Carver College of Medicine, University of Iowa, Iowa City, IA 52242, USA; Fraternal Order of Eagles Diabetes Research Center, University of Iowa, Iowa City, IA 52242, USA; Endocrinology Section, Iowa City Veterans Affairs Medical Center, Iowa City, IA 52246, USA; Department of Internal Medicine, Carver College of Medicine, University of Iowa, Iowa City, IA 52242, USA; Fraternal Order of Eagles Diabetes Research Center, University of Iowa, Iowa City, IA 52242, USA; Endocrinology Section, Iowa City Veterans Affairs Medical Center, Iowa City, IA 52246, USA; Department of Internal Medicine, Carver College of Medicine, University of Iowa, Iowa City, IA 52242, USA; Fraternal Order of Eagles Diabetes Research Center, University of Iowa, Iowa City, IA 52242, USA; Endocrinology Section, Iowa City Veterans Affairs Medical Center, Iowa City, IA 52246, USA; Department of Internal Medicine, Carver College of Medicine, University of Iowa, Iowa City, IA 52242, USA; Fraternal Order of Eagles Diabetes Research Center, University of Iowa, Iowa City, IA 52242, USA; Endocrinology Section, Iowa City Veterans Affairs Medical Center, Iowa City, IA 52246, USA; Department of Internal Medicine, Carver College of Medicine, University of Iowa, Iowa City, IA 52242, USA; Fraternal Order of Eagles Diabetes Research Center, University of Iowa, Iowa City, IA 52242, USA; Endocrinology Section, Iowa City Veterans Affairs Medical Center, Iowa City, IA 52246, USA; Department of Internal Medicine, Carver College of Medicine, University of Iowa, Iowa City, IA 52242, USA; Fraternal Order of Eagles Diabetes Research Center, University of Iowa, Iowa City, IA 52242, USA; Endocrinology Section, Iowa City Veterans Affairs Medical Center, Iowa City, IA 52246, USA; Department of Internal Medicine, Carver College of Medicine, University of Iowa, Iowa City, IA 52242, USA; Fraternal Order of Eagles Diabetes Research Center, University of Iowa, Iowa City, IA 52242, USA; Endocrinology Section, Iowa City Veterans Affairs Medical Center, Iowa City, IA 52246, USA; Department of Internal Medicine, Carver College of Medicine, University of Iowa, Iowa City, IA 52242, USA; Fraternal Order of Eagles Diabetes Research Center, University of Iowa, Iowa City, IA 52242, USA; Endocrinology Section, Iowa City Veterans Affairs Medical Center, Iowa City, IA 52246, USA

**Keywords:** lipid droplets, cAMP-dependent kinase, peroxisome proliferator-activated receptor alpha, insulin receptor substrate 2

## Abstract

Perilipin 5 (PLIN5) is a lipid droplet (LD) protein highly expressed in cells that actively oxidize fatty acids. Previous in vitro studies have revealed that PLIN5 phosphorylation (p-PLIN5) at serine 155 by protein kinase A is critical for transcriptional regulation of PPARa target genes by which PLIN5 adapt cells for fatty acid oxidation. We aim to determine the extent of p-PLIN5 in vivo and the consequence of impaired PLIN5 phosphorylation in the liver by using a whole-body knock-in of phosphorylation-resistant PLIN5 (SA/SA) in mice. Plin5 phosphorylation at S155 was increased in the liver LD fraction of fasted mice compared with that of fed mice by mass spectrometry (*P* < .05). Quantitative polymerase chain reaction of key lipid metabolism genes did not differ between wild-type and SA/SA liver upon fasting in both young and old males. Young SA/SA female mice showed a small but significant reduction in the expression of *Ppara* and *Cpt1a* genes in the liver after overnight fasting. Male SA/SA mice had higher fasting blood glucose (*P* < .05) without a difference in body weight, serum insulin, or serum lipids. IRS2 was reduced in the liver of fasted male SA/SA mice (*P* < .05). PLIN5 S155 phosphorylation has a limited impact on the upregulation of hepatic lipid metabolism genes important for fasting response in vivo in females and is largely dispensable in males. Impaired phosphorylation also had little effect on serum lipids or liver triglycerides. However, old SA/SA mice showed decreased IRS2 expression in the liver, which may contribute to glucose intolerance in SA/SA male mice.

The lipid droplet (LD) is an organelle that serves as a hub for intracellular lipid metabolism with its ability to store neutral lipids and release lipids in a temporally and spatially regulated manner (1). Five members of the perilipin family of protein (PLIN1-5) reside on the surface of LDs and confer the regulation on lipid metabolism, each with a unique expression pattern and molecular characteristics (2, 3). PLIN1 is the best studied PLIN whose expression is restricted to adipocytes and adrenocortical cells. PLIN1 allows massive accumulation of triglycerides (TGs) in adipocytes at the basal status. Upon activation of protein kinase A (PKA), such as during fasting, adipocyte PLIN1 facilitates rapid degradation of TGs by lipolysis to release fatty acids (FAs) into circulation for the use in extra adipose tissues (3). PLIN5 is highly expressed in cells and conditions that heavily depend on fatty acid oxidation (FAO) such as brown adipocytes, cardiomyocytes, oxidative skeletal muscle cells, hepatocytes upon fasting, and pancreatic islets upon fasting (4, 5). Studies primarily performed in cell culture models have revealed that PLIN5 has molecular characteristics beneficial for adaptation to high levels of FAO. At the basal status, PLIN5 prevents lipolysis by sequestrating adipose triglyceride lipase (PNPLA2) from its coactivator ABHD5, thus expanding the TG pool (6-8). Upon PKA activation, PLIN5 promotes lipolysis by PNPLA2 in part by increasing the interaction between PLIN5 and PNPLA2 (9-11). Mouse PLIN5 contains 1 PKA consensus sequence (RRXS) at amino acid S155 that is RRWS (12), which is considered to be necessary to upregulate lipolysis under PKA activation (10). In addition, PLIN5 phosphorylation at S155 by PKA triggers nuclear translocation of PLIN5 that is proposed to transport monounsaturated FA to nuclei and to activate peroxisome proliferator– activated receptor-gamma coactivator (PGC1a) through deacetylation catalyzed by Sirtuin 1 (SIRT1), collectively upregulating the expression of PPARa target genes important for FAO, mitochondrial mass/function, autophagy, and inflammation (7, 8, 11, 13, 14). The importance of lipolysis in the regulation of PPARa target genes is also demonstrated in mouse models in which *Pnpla2* downregulation in cardiomyocytes and hepatocytes reduced the expression of PPARa target genes (15, 16). The transcriptional change mediated by lipolysis and PLIN5 is considered to adapt cells to the increased demand of FAO and stress associated with FAO. Thus, PKA responsiveness of PLIN5 appears to be critical for upregulation of lipolysis and the transcriptional regulation in cells under high demand of FAO. However, the impact of PLIN5 phosphorylation in vivo remains less defined since most studies of PLIN5 phosphorylation utilized in vitro models with pharmacological stimulation of PKA (9, 13, 17). Two studies reported phenotypes of mice expressing phosphorylation-resistant PLIN5 in cardiomyocytes and hepatocytes, but both compared wild-type (WT) and phosphorylation-resistant PLIN5 overexpressed under exogenous promoters, and the impact of PLIN5 phosphorylation on hepatic PPARa gene expression was not directly compared between WT and phosphorylation-resistant PLIN5 expressed at physiological levels (8, 11). Here, we address the extent of PLIN5 phosphorylation in vivo and the consequence of impaired phosphorylation using a model in which phosphorylation-resistant PLIN5 was expressed under the endogenous promoter (SA/SA mice). The liver upon fasting was tested as a physiological condition when PPARa activation plays a critical role in switching lipid metabolism to increase FAO (18). Also, PKA-dependent PLIN5 phosphorylation in the liver is expected to increase during fasting due to the rise of cAMP (19, 20). We demonstrated that the total PLIN5 protein level is increased during fasting in the liver with the proportional increase of phosphorylated PLIN5. SA/SA knock-in mice that replaced S155 PLIN5 with alanine showed significant reduction in PLIN5 phosphorylation in the liver upon fasting, supporting that S155 is the major phosphorylation site of PLIN5. However, liver TGs, serum lipids, serum ketone, and the expression of hepatic lipid metabolism genes that are known to be regulated by PPARa during fasting were not altered in male SA/SA mice upon fasting. Female SA/SA mice showed mild reduction in a small fraction of hepatic lipid metabolism genes. As for glucose homeostasis, old male SA/SA mice showed mild glucose intolerance that was associated with reduced expression of IRS2 in the liver.

## Materials and Methods

### Cell Culture

Adenovirus expressing WT mouse PLIN5 (Ad-PLIN5) and PLIN5 in which serine 155 was mutated to alanine (SA) were created by Vector Biolabs (Philadelphia, PA) with confirmation of cDNA sequence. Hepatocytes from C57BL/6J mice were provided by Triangle Research Labs (Charlottesville, VA). Hepatocytes were transduced in suspension with 100 plaque forming units (pfu) per cell of Ad-PLIN5 for 1 hour in serum-free Dulbecco’s modified Eagle’s medium; (DMEM) containing 22.2 mM glucose, plated at 2.5 × 10^5^ cells/well in a 6-well plate, and cultured overnight in the presence of 10% fetal bovine serum DMEM at 37 °C in 5% CO_2_. Then, Ad-PLIN5-transduced hepatocytes were incubated for 24 hours in DMEM containing 9 mM glucose plus 0.5 mM oleic acid conjugated to 1% FA-free albumin to promote LD formation. The next day, cells were incubated in serum-free 9 mM glucose DMEM for 5 hours, treated with and without 20 μM H89 dihydrochloride (Cell Signaling, Beverly, MA) for 30 minutes followed by incubation with 1 mM 8-bromoadenosine 3′,5′-cyclic monophosphate (8-Br-cAMP, Sigma, St Louis, MO) for 15 minutes, washed with cold phosphate-buffered saline, snap frozen by submerging wells in liquid nitrogen, and scraping into cold immunoprecipitation (IP) buffer (249 μM mannitol, 0.5% triton-X, 50 mM Tris HCl pH 7.5 supplemented with protease and phosphatase inhibitor cocktails [Sigma]) for further analysis. AML12 cells (CRL-2254, American Tissue Culture Collection, Manassas, VA) were maintained in DMEM:F12 medium supplemented with 10% fetal bovine serum, 10 μg/mL insulin, 5.5 μg/mL transferrin, 5 ng/mL selenium, and 40 ng/mL dexamethasone. AML12 cells were transduced with Ad-PLIN5 or Ad-SA PLIN5 as in hepatocytes, loaded with 0.2 mM oleic acid starting from the next day, and harvested on the third day as for hepatocytes after 1 hour incubation with or without 8-Br-cAMP.

### Animal Studies

Experiments were approved by and performed in accordance with the Institutional Animal Care and Use Committee guidelines of University of Iowa. This includes monitoring of health of mice, care to minimize pain, and euthanasia in accordance with guideline by American Veterinary Medical Association. Mice were housed 5/cage in 12 hours light–dark cycle at 22 °C, allowed free access to water, and fed regular rodent chow (7319 Teklad global diet) except during fasting experiments. Female leptin mutant *ob/ob* mice and WT mice (BL6J) in C57BL/6J background were obtained from Jackson Laboratory (Bar Harbor, ME). For some experiments, male WT C57BL/6NJ (BL6NJ) mice from Jackson Laboratory were used. The treadmill exercise test was performed as previously described (21). In brief, mice after acclimation were placed on the treadmills at a 10° angle and ran at the starting speed of 4 m/min with 2 m/min increase in the speed every 10 minutes for 70 minutes. After 70 minutes, the speed was increased by 4 m/min every 10 minutes until exhaustion, determined by the mice being unable to leave the treadmill shock grids for 10 consecutive seconds. Shock grids were set to irritate but not harm the mice. Mice in which serine at 155 (AGT) of PLIN5 was replaced with alanine (GCC) by CRISPR-Cas9 gene editing were created at the Genome Editing Core Facility at University of Iowa (SA/SA mice). The correct replacement of the targeted sequence was confirmed by DNA sequencing in a founder mouse. The genotype of subsequent offspring was performed as in the supplemental material (22). The young male mouse group was harvested at 3 to 4 months of age, old male mouse group at 1 year of age, and female mouse group at 4 to 6 months of age. At the time of harvest, the liver and the heart were snap frozen in liquid nitrogen and stored at −80 °C until analyses. A part of the liver was fixed in 10% formalin, embedded in paraffin, and sectioned for hematoxylin and eosin staining.

### Lipid Droplet Semipurification

**Figure 1.**
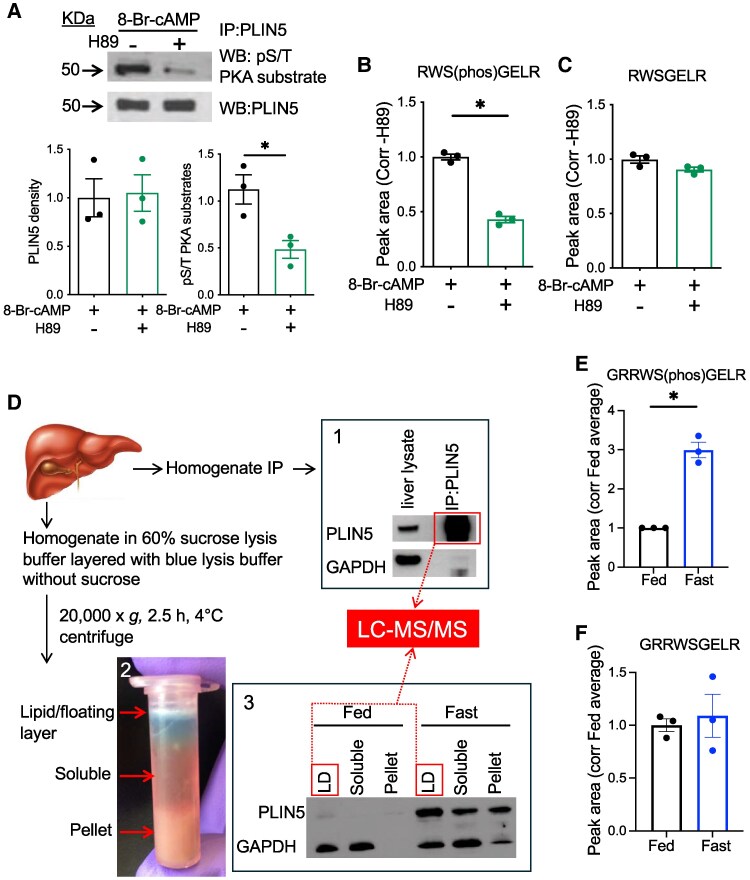
S155 PLIN5 phosphorylation detected by mass spectrometry is increased in the LD fraction of liver from fasted mice. (A) Western blot (WB) of PLIN5 immunoprecipitated (IP) from hepatocyte lysate overexpressing wild type (WT) PLIN5 and treated with 8-Br-cAMP ± H89 was performed for indicated antibodies. Representative images and densitometry data are shown. (B, C) Spectral peak areas of PLIN5 peptides RWS(phos)GELR (B) and RWSGELR (C) were obtained by liquid chromatography tandem mass spectrometry (LC-MS/MS) for tryptic peptides of (A). (D) Schematic diagram of PLIN5 enrichment from WT mouse liver homogenate and LC-MS/MS analysis. 1. WB of PLIN5 and GAPDH comparing whole liver lysate and PLIN5 IP from the fast liver. 2. Photo depicts the separation of lipid/floating layer, soluble layer, and pellet after the single centrifugation step as in “Material and Methods.” 3. WB compared PLIN5 and GAPDH levels in the 3 fractioned layers from the livers of fed and fasted mice. Fractions applied to LC-MS/MS are indicated by boxes. (E, F) Spectral peak areas for PLIN5 peptides GRRW(phos)SGELR (E) and GRRWSGELR (F) obtained from 67 µg of protein from fed and fast liver LD fractions in 3. Data are means ± SEM; n = 3. *P < .05 by the Student t test.

LDs were semipurified by a single centrifugation protocol published by Harris et al (23) with the following modifications (Fig. 1D): 400 to 600 mg of the left lobe of the liver snap frozen upon harvest was transferred to ice-cold 60% sucrose lysis buffer (23), crushed with a pestle, and incubated on ice for 10 minutes with vortexing every 5 minutes. After adding lysis buffer without sucrose for a final volume of 1 mL, liver samples were incubated on ice for additional 10 minutes with vortexing every 5 minutes. Liver samples were then homogenized with a handheld homogenizer and incubated on ice for 15 minutes with vortexing every 5 minutes; 300 μL of lysis buffer with blue food color dye was layered on top of homogenate and centrifuged at 20 000*g* for 2.5 hours at 4 °C, which enriches LDs in a floating layer on top of the blue dye layer (Fig. 1D2). The LD layer was transferred to a new tube for further analysis.

### Immunoprecipitation and Western Blotting

For IP, tissues and cells were lysed in IP buffer. Protein concentrations were measured with Bio-Rad DC Protein Assay (Bio-Rad, Hercules, CA). PLIN5 IP used guinea pig anti-PLIN5 antibody (AB_2909602, Progen Biotechnik, Heidelberg, Germany) and Pierce's Classic Immunoprecipitation Kit (Thermo Scientific, Waltham, MA) according to the manufacturer's instructions. Western blots were performed using 20 to 40 μg/lane of protein and detected by chemiluminescence or Odyssey CLx imaging system (Licor, Lincoln, NE). Densitometric analyses were performed with Image J. Antibodies and dilution used are in Table S1 (24). For PLIN5 in-gel digestion, 67 μg of protein from LD fraction was run on a Bis-Tris gel. For separation of p-PLIN5, SuperSep Phos-tag (50 µmol/L) 12.5% precast gel (Wako Chemical, Richmond, VA) was used. A 12- to 15-μg bolus of liver lysate per lane was run on the gel and the gel was washed prior to transfer following manufacturer's instructions. Proteins were transferred onto a nitrocellulose membrane and blocked in phosphate-buffered saline containing 0.1% Tween 20 and 3% bovine serum albumin prior to antibody treatment.

### PLIN5 In-Gel Reduction, Alkylation, Denaturation and Trypsin Digestion

The gel stained with Page Blue was destained and sequentially washed with buffers containing 50 mM ammonium bicarbonate, 50% acetonitrile, and 80% acetonitrile. The bound proteins were reduced with 1 mL of 40 mM dithiothreitol for 25 minutes at 56 °C. The gels were rinsed with 1 mL of 50 mM ammonium bicarbonate buffer and the reduced proteins were alkylated with 1 mL of 50 mM iodoacetamide for 30 minutes at 25 °C in the dark with constant mixing. The iodoacetamide was discarded and the gel-bound proteins were digested with 0.5 mL of 20 ng/µL trypsin (Promega, Madison, WI) in 50 mM ammonium bicarbonate buffer at 37 °C with constant mixing for 12 hours. After digestion, the tryptic fraction released into 50 mM ammonium bicarbonate buffer was collected, and the gels were washed with 50 mM ammonium bicarbonate to collect any remaining tryptic peptides. The ammonium bicarbonate buffer containing the tryptic peptides was dried using a Speed-Vac apparatus and stored at 4 °C prior to mass spectrometric analysis.

The dried samples were dissolved with 20 µL of 0.1% formic acid in water. A 2-µL bolus of each sample was analyzed by liquid chromatography tandem mass spectrometry (LC-MS/MS) using a Q-Exactive mass spectrometer (Thermo Fisher) with an Easy NanoLC-1000 system as in the supplemental material (22).

### Colorimetric Assays

Saphenous blood or cardiac blood collected at harvest was centrifuged at 2000*g* for 10 minutes at room temperature to obtain serum. TGs (TR22421 from ThermoFisher), β-hydroxybutyrate (Stanbio, Boerne, TX), nonesterified FAs (Wako Chemicals), and mouse insulin (AB_3065112, Alpco, Salem, NH) were measured according to manufacturer's protocols. Liver TG was measured using an aforementioned TG colorimetric assay after Folch extraction and values were corrected for protein contents (25).

### mRNA and Quantitative Polymerase Chain Reaction

Total RNA from liver tissues (50-100 mg) was prepared using Qiagen RNeasy kit (Qiagen, Valencia, CA) according to the manufacturer's protocol and cDNA was synthesized as published (26). Gene expression was assessed as previously described (26) using ABI TaqMan commercial primers (Applied Biosystems). Results were expressed using mouse PPIB as a reference gene.

### Bulk RNA-sequence of the Liver

RNA was extracted from the liver using TRIzol Reagent (Invitrogen, Carlsbad, CA) followed by RNA Clean & Concentrator-5 (Zymo Research, Irvine, CA) kit with DNase I treatment according to manufacturer instructions. An RNA integrity number (RIN) was measured by RNA ScreenTape (Agilent Technologies, Santa Clara, CA), and RNA with RIN above 7.2 was used. Gene expression profiling by bulk RNA-seq was performed by the University of Iowa Genomics Division as in the supplemental material (22). The raw FASTQ files and associated metadata have been made available for download at GEO accession GSE275608.

### Glucose Homeostasis

A glucose tolerance test (GTT) was done after overnight fasting by loading 1 mg/g BW glucose intraperitoneally and tail blood glucose was measured at indicated time using a handheld glucometer. An insulin tolerance test (ITT) was performed after 6 hours of fasting using 0.75 mU/g bodyweight regular insulin intraperitoneally and glucose was measured as in a GTT.

### Statistical Analysis

Data are presented as mean ± standard error of mean (SEM) unless otherwise stated in the figure legends. Differences of numeric parameters between 2 groups were assessed with Student t-tests. Welch correction was applied when variances between 2 groups were significantly different by F test using Prism 10 (GraphPad, La Jolla, CA). Multiple group comparisons used 1-way analysis of variance (ANOVA) with post hoc test as indicated. When 2 categorical variants exist, 2-way a ANOVA test with post hoc test was performed. *P* < .05 was considered statistically significant.

## Results

### Fasting Increases PLIN5 Phosphorylated at S155 in the Mouse Liver

While S155 has been shown to be phosphorylated in a PKA-dependent manner in vitro, phosphorylation of PLIN5 at S155 in response to physiological stimuli has not been shown in the liver (11, 13). To establish a protocol to detect S155 PLIN5 by LC-MS/MS, primary hepatocytes overexpressing PLIN5 were treated acutely with a cAMP analog, 8-Br-cAMP in the presence or absence of a PKA inhibitor H89. PLIN5 was enriched by IP and analyzed by Western blot (Fig. 1A) and LC-MS/MS (Fig. 1B and 1C) in parallel. In Western blot, H89 markedly reduced the phospho-PKA substrate signal of PLIN5 IP in support of PKA dependency of PLIN5 phosphorylation (Fig. 1A). LC-MS/MS of these IP samples yielded RWS(phos)GELR as a dominant tryptic peptide containing S155 of PLIN5 in primary hepatocytes (Fig. 1B; Fig. S1A) (27). While abundance of RWSGELR peptide was similar in both groups, RWS(phos)GELR peptide was reduced in H89-treated hepatocytes showing good agreement with Western blot (Fig. 1B-1C). We then analyzed PLIN5 enriched by IP of the liver lysate from male WT BL/6NJ mice fasted overnight by LC-MS/MS (Fig. 1D1). Fasting caused 11% weight loss, 51% reduction of blood glucose, 701% increase of serum beta-hydroxybutyrate, and 225% increase of PLIN5 protein by Western blot of liver lysate, as expected (Fig. S1B-E) (4, 27). Tryptic peptides of PLIN5 IP samples from the fasted mouse liver analyzed by LC-MS/MS showed that GRRWS(phos)GELR is the major peptide that includes S155 in the liver. Then, we moved to the quantitative comparison of GRRWS(phos)GELR peptide in the fasted and fed liver. We chose to utilize a LD fraction semipurified from liver homogenate due to a significant difference in total PLIN5 protein levels that may not allow the proportional recovery of PLIN5 after IP (Fig. 1D2). PLIN5 was primarily detected in the LD fraction in both fed and fasted liver and was much more abundant in the fasted liver (Fig. 1D3). When an equal amount of protein was loaded, the area of the spectral peak for GRRWS(phos)GELR peptide in the LD fraction was significantly increased in the fast liver compared with fed liver (Fig. 1E), while that for GRRWSGELR did not differ (Fig. 1F). Thus, LC-MS/MS indicated that fasting increases phosphorylation of PLIN5 at S155 in the liver LD fraction of fasted mice.

### Phos-tag Gel Allows Quick Separation of Nonphosphorylated and Phosphorylated PLIN5

**Figure 2.**
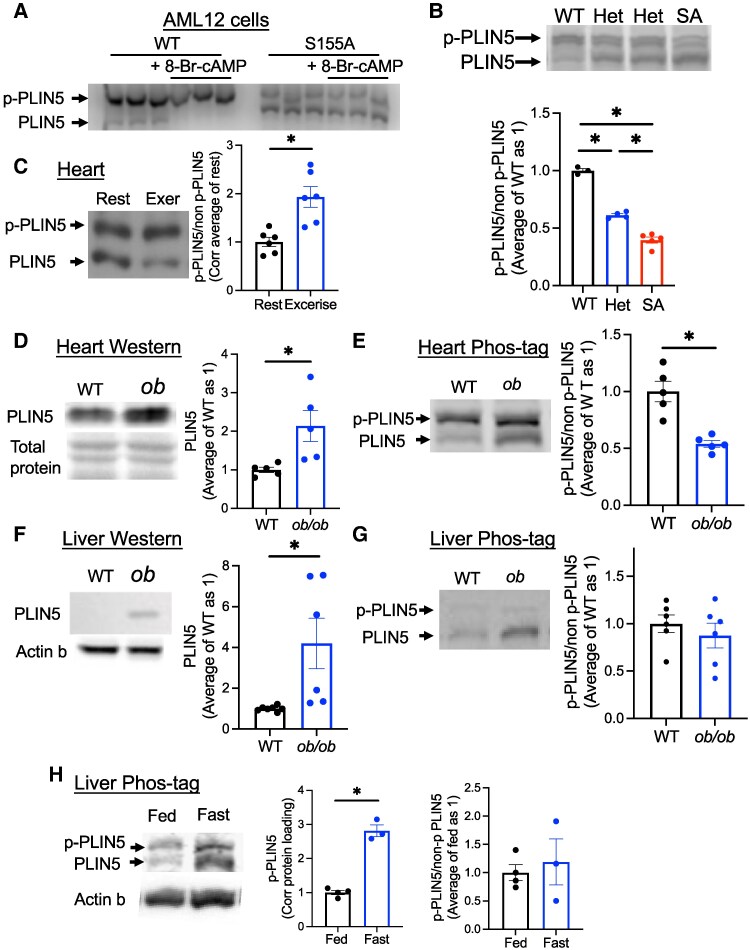
Phos-tag gel allows the detection of p-PLIN5. (A) Cell lysate from AML12 cell overexpressing wild type (WT) or S155A PLIN5 and treated with or without 8-Br-cAMP was run on Phos-tag gel and immunoblotted by anti-PLIN5 antibody. Representative image of 3 experiments. (B) Phos-tag gel of the liver from fasted 1-year-old male WT, SA het (het) and SA/SA (SA) mice assessed the ratio of phosphorylated (p-PLIN5) and nonphosphorylated (non-p-PLIN5). n = 3-5. (C) Phos-tag gel assessed p-PLIN5 and non-p-PLIN5 in the heart of WT mice with (Exer) and without (Rest) treadmill exercise. n = 6. (D-G) The heart (D, E) and the liver (F, G) lysates from female WT and *ob/ob* mice were analyzed for PLIN5 by regular Western blot (D, F) and Phos-tag gel (E, G), n = 5-7. Western blot of PLIN5 was normalized by total protein staining for the heart (D) and actin b for the liver (F). (H) Phos-tag gel assessed the abundance of p-PLIN5 and non-p-PLIN5 in the total liver lysate of fed or overnight fasted male mice. n = 3-4. (B-H) Representative gels and densitometry data are shown. Data are mean ± SEM. (B) *P < .05 by 1-way ANOVA with the Sidak multiple comparison test. (C-H) *P < .05 by the Student t test.

While mass spectrometry provides measurement of p-PLIN5 specifically at S155, it is time and resource consuming. Phos-tag gel contains the Phos-tag molecule that specifically binds to phosphorylated proteins and slows their migration during sodium dodecyl sulfate polyacrylamide gel electrophoresis (28). We tested whether Phos-tag gel can be utilized to differentiate nonphosphorylated from phosphorylated forms of S155 PLIN5. First, AML12 cells expressing WT and SA PLIN5 were stimulated by 8-Br-cAMP, and cell lysates were run on Phos-tag gel. As shown in Fig. 2A, WT PLIN5 showed predominance in the top band at baseline with disappearance of the bottom band in the presence of 8-Br-cAMP. In contrast, SA PLIN5 showed predominance of the bottom band at baseline that did not change in the presence of 8-Br-cAMP. Moreover, Phos-tag gel showed a stepwise reduction of p-PLIN5 in the liver lysate from PLIN5^SA/+^ (SA het) and PLIN5^SA/SA^ knock-in (SA/SA) male mice fasted overnight compared with PLIN5^+/+^ (WT) mice, confirming that S155 is the major phosphorylation site of PLIN5 in the liver PLIN5 in vivo (Fig. 2B). Thus, Phos-tag gel can detect changes in PLIN5 phosphorylation at S155 both in cell culture and mouse models (Fig. 2A and 2B).

### Phos-tag Gel Detects the Change in PLIN5 Phosphorylation In Vivo in the Liver and the Heart

We then asked whether the proportion of p-PLIN5 can be altered in conditions that increase FAO or PLIN5 expression in vivo. During exercise, the heart activates sympathetic tone that increases intracellular cAMP and FAO (29). The heart harvested from WT mice run on a treadmill (run distance 1149 ± 100 m, mean ± SEM, n = 6) showed an increased proportion of p-PLIN5 (Fig. 2C). The validity of the exercise was supported by the increase in blood lactate levels (Fig. S2A) (27). Next, we tested whether the proportion of p-PLIN5 is altered when obesity increases PLIN5 protein in the liver and heart (Fig. 2D-2G) using *ob/ob* mice (body weight and blood glucose in Fig. S2B, C, (27)). Western blot showed the increase of PLIN5 in both the liver and the heart of *ob/ob* mice compared with WT mice (Fig. 2D and 2F). The proportion of p-PLIN5 was reduced in the heart but not altered in the liver of *ob/ob* mice compared with WT mice (Fig. 2E and 2G). During fasting, FA released by adipose lipolysis is distributed to the liver and the heart for energy use. In the liver from fasted WT mice, p-PLIN5 corrected for protein was increased in Phos-tag gel (Fig. 2H). However, there was no statistically significant change in the proportion of p-PLIN5, as nonphosphorylated PLIN5 in total liver lysate was also increased upon fasting (Fig. 2H). Combined with LC-MS/MS data (Fig. 1E and 1F), p-PLIN5 is increased during fasting, especially in LDs in the liver, but this may be proportional to the increase in total PLIN5 content in the liver based on Fig. 2H. Fasting did not increase the proportion of p-PLIN5 in the heart of WT mice either (Fig. S2 (27)). Collectively, Phos-tag gel revealed that the extent of p-PLIN5 varies depending on tissues and context, with more dynamic changes in the heart than in the liver.

### Parameters of Lipid Metabolism and the Expression of Hepatic PPARa Target Genes Important for Fasting Response Showed Limited Changes in WT and SA/SA Mice

**Figure 3.**
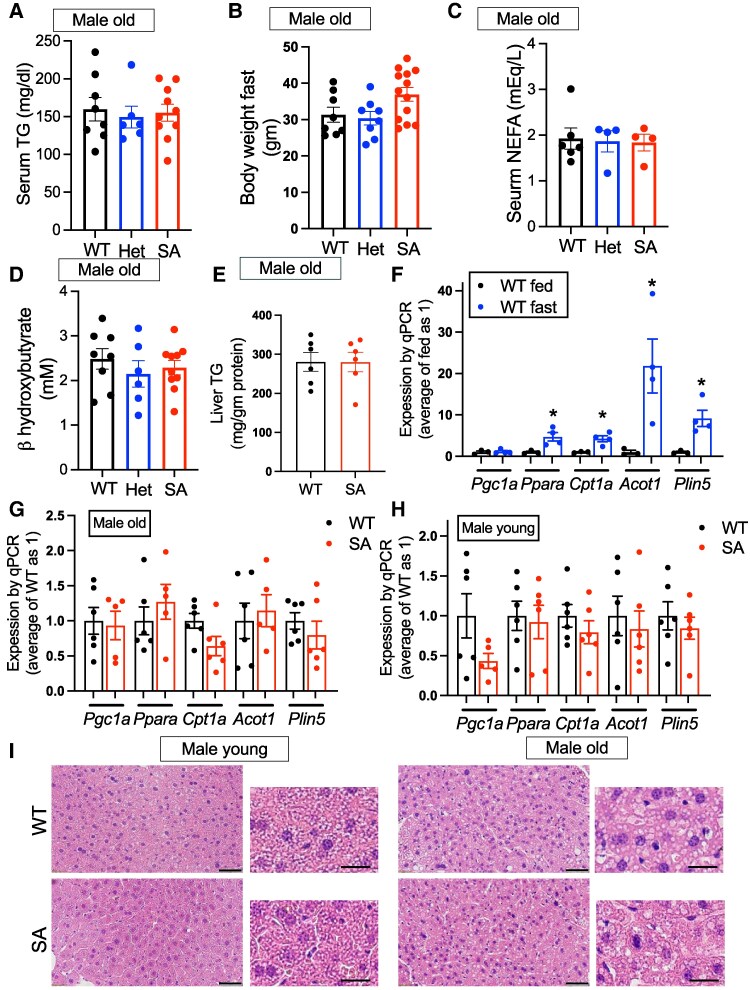
Serum chemistry and hepatic lipid metabolism gene expression in fasted PLIN5 SA/SA male mice. (A) Body weight, (B) serum TG, (C) serum nonesterified fatty acids (NEFAs), and (D) serum β-hydroxybutyrate of 1-year-old male mice (male old) after overnight fasting. Each dot represents 1 mouse. n = 6-8 (WT), 4-8 (het), and 4-13 (SA). (E) TG contents in the liver corrected for liver protein (n = 6). (F-H) qPCR compared the expression of genes in lipid metabolism in the liver of (E) WT mice fasted overnight (n = 3) vs fed ad libitum (n = 4), (F) 1-year-old WT (n = 6) vs SA mice fasted overnight (n = 5-6), and (H) 3-month-old WT (n = 6) vs SA male mice (male young) fasted overnight (n = 5-6). (I) Representative hematoxylin and eosin staining of the liver after overnight fast for old and young WT and SA male mice. Low magnification on the left (scale bar, 50 μm) and high magnification on the right (scale bar, 20 μm). Data are mean ± SEM. *P < .05 by the Student t test.

**Figure 4.**
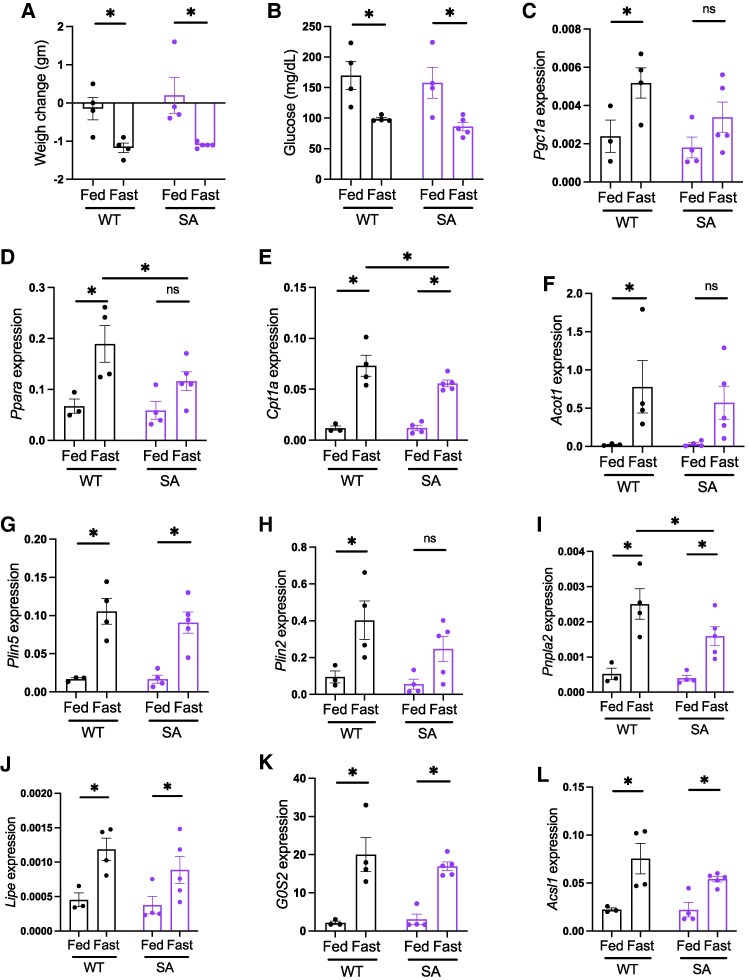
Hepatic lipid metabolism gene expression in young fed and fasted PLIN5 SA/SA female mice. Four-month-old female WT and SA mice were either fed ad libitum or fasted overnight. (A) Change in body weight and (B) blood glucose after overnight. (C-L) qPCR compared the expression of genes in lipid metabolism in the liver. Expression levels of target genes were corrected by *Ppib* expression. Each dot represents 1 mouse. Data are mean ± SEM. *P < .05, ns; not significant by the uncorrected Fisher LSD test of 2-way ANOVA.

One proposed consequence of p-PLIN5 is upregulation of PPARa target genes by increasing lipolysis and activating Sirt1/PGC1a pathway (13, 14). PPARa is the master transcriptional regulator mediating dramatic changes in the expression of lipid metabolism genes during fasting in the liver (18). Previously, a subset of the PPARa target lipid metabolism gene was shown to be transcriptionally regulated by lipolysis and/or PLIN5 phosphorylation in cultured cells (13, 14). Thus, we used SA/SA mice to assess whether S155 PLIN5 phosphorylation is necessary for expression of PPARa target genes during fasting in the liver. Body weight, serum TGs, serum nonesterified FAs (NEFAs), and serum b-hydroxybutyrate did not differ between WT, het, and SA/SA knock-in mice after fasting in 1-year-old male mice (Fig. 3A-3D) or 4-month-old female mice (Fig. S3A-D (27)). Liver TG contents were not altered in 1-year-old male mice (old male group) or female mice after fasting (Fig. 3E; Fig. S3E (27)). Then, we first chose *Pgc1a*, *Ppara*, *Cpt1a*, *Acot1*, and *Plin5* as lipid metabolism genes of interest as their expression was previously shown to be regulated by p-PLIN5 (13, 14). While the expression of *Ppara* can be regulated by glucocorticoid and *Cpt1a* by CREB3L3 during fasting in the liver (18), *Acot1* and *Plin5* are regulated by PPARa (30, 31). The expression of *Ppara*, *Cpt1a*, *Acot1*, and *Plin5* was markedly upregulated after overnight fasting in the liver of male WT mice, as expected (Fig. 3F). Surprisingly, there was little difference in the expression of these genes between WT and SA mouse liver after fasting in the old male group (Fig. 3G). The liver of 3- to 4-month-old male mice (young male group) after fasting did not show a difference in *Ppara*, *Cpt1a*, *Acot1*, and *Plin5* expression between WT and SA/SA either (Fig. 3H). Although *Pgc1a* appears to be expressed less in SA/SA liver of young male mice, there was not statistical significance compared with WT (Fig. 3H). Body weight and liver weight did not differ between WT and SA/SA for young male mice (Fig. S3F, G (27)). Hematoxylin and eosin staining showed steatosis typical of fasting in the liver of young male mice while the formation of larger LDs was prominent in the liver of 1-year-old male mice. However, there was not a clear difference between liver histology of WT and SA/SA mice (Fig. 3I).

As fasting response is reported to be more pronounced in females (32), we next tested whether 4-month-old SA/SA female mice show a difference in hepatic lipid metabolism gene expression upon fasting compared with WT mice. In this cohort, we included ad libitum fed mice of both genotypes. There was expected reduction in weight and glucose after an overnight fast to a similar extent in both WT and SA/SA female mice (Figs. 4A and 4B). For the 5 lipid metabolism genes tested in male mice, a small decrease of fasting response in SA/SA liver was implicated in 4 genes in post hoc tests of 2-way ANOVA (Fig. 4C-4F). When fasting increased expression of all 5 genes in WT liver, *Pgc1a*, *Ppara*, and *Acot1* did not reach statistical significance between fed and fasted liver in SA/SA. *Ppara* and *Cpt1a* expression were lower in SA liver than in WT liver upon fasting. Additionally, 2 other genes known to be upregulated upon fasting (*Plin2* and *Pnpal2*) showed a difference between WT and SA/SA (Fig. 4G and 4H). However, we did not see the effect of SA mutation for other lipid metabolism genes reported to be regulated by PLIN5 in other tissues and cultured cells. The expression of *Lipe* and *G0s2* was reduced in the heart of mice overexpressing SA PLIN5 compared with mice without overexpression (8). *Acsl1* is a PPARa target gene whose expression is reduced in the liver treated with PLIN5 antisense oligonucleotide (14). These 3 genes were similarly upregulated by fasting in the liver of WT and SA female mice (Fig. 4I-4K). *Lipe*, *G0s2*, and *Acsl1* were not differentially expressed in the liver of fasted old or young SA/SA mice either (Fig. S3H, I (27)).

Overall, parameters of lipid metabolism including blood chemistry and liver TG levels showed little changes in SA/SA mice after fasting regardless of age and genders. There was subtle reduction in a part of lipid metabolic genes only in the liver of female mice upon fasting. However, a 2-way ANOVA test showed that an effect of genotype is negligible compared with the effect of fasting in these genes in female mouse liver (Fig. S3J (27)).

### RNA Sequencing of Female SA/SA Liver Showed Limited Number of Differentially Expressed Genes

**Figure 5.**
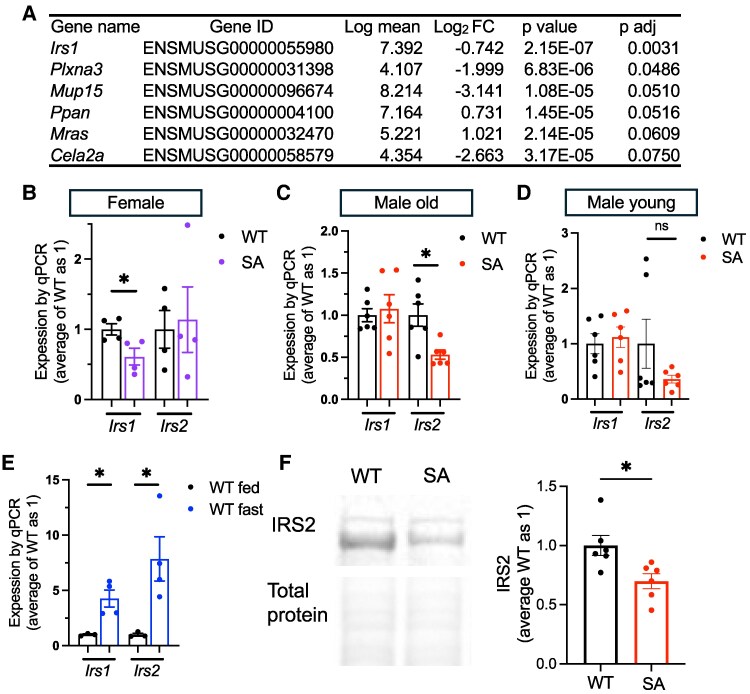
RNA sequencing of SA/SA liver identified differential expression of *Irs1* and *Irs2* in the liver. (A) The list of genes differentially expressed in the liver of female SA/SA (SA) mice compared with WT mice in RNA sequencing. FC, fold change. (B-D) qPCR compared the expression of *Irs1* and *Irs2* in the liver of (B) overnight fasted WT and SA female mice, n = 4, (C) overnight fasted 1-year-old WT and SA male mice, n = 6, (D) overnight fasted 3-month-old WT and SA male mice, n = 6, and (E) overnight fasted and ad libitum fed WT male mice, n = 3-4. (F) Western blot compared IRS2 and total protein levels in the livers of fasted WT and SA male mice. n = 6. Data are mean ± SEM. *P < .05, ns; not significant by the Student t test.

**Figure 6.**
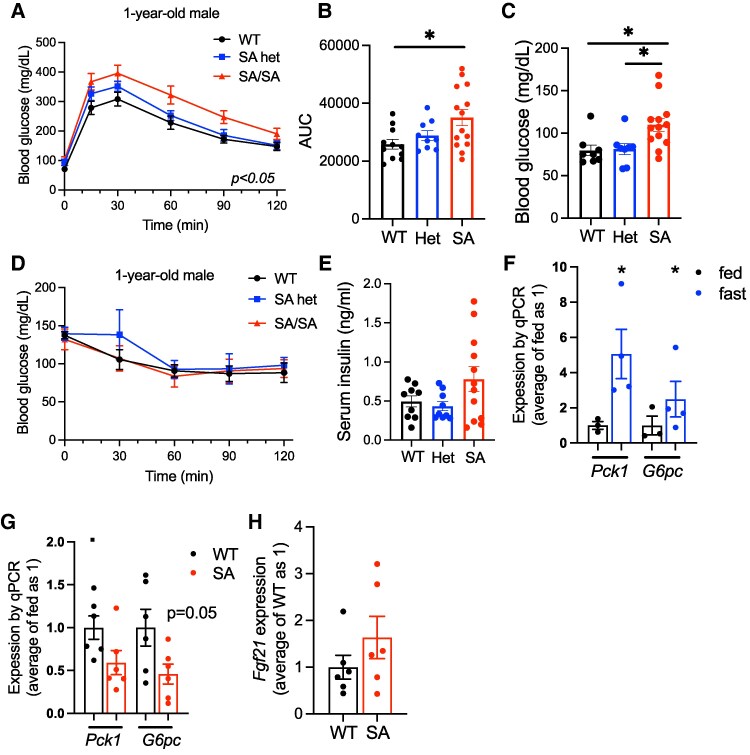
Glucose homeostasis and the expression of gluconeogenesis genes in the liver of 1-year-old PLIN5 SA/SA male mice. (A) Intraperitoneal glucose tolerance test compared male WT, SA het (het), and SA/SA (SA) mice at 1 year of age. (B) Area under the curve (AUC) of (A). (C) Fasting glucose of (A). (D) Intraperitoneal insulin tolerance test compared male WT, het, and SA mice at 1 year of age. (E) Serum insulin of 1-year-old male mice after overnight fasting. (A-E) n = 7-11 (WT), 3-9 (het), and 5-14 (SA). (F, G) qPCR compared the expression of *Pck1* and *G6pc* (F) in the liver of WT mice fasted overnight vs fed ad libitum, n = 3-4, and (G) 1-year-old WT vs SA male mice fasted overnight, n = 5-6. (H) qPCR compared the expression of *Fgf21* in the liver of 1-year-old WT vs SA mice fasted overnight. n = 6. Data are mean ± SEM. (A) Two-way ANOVA test. (B, C) *P < .05 by 1-way ANOVA with the Sidak multiple comparison test. (F, G) *P < .05 by the Student t test.

We next performed RNA sequencing to identify genes differentially regulated by the loss of S155 phosphorylation in an unbiased manner using the liver of female WT and SA mice after overnight fasting. RNA sequencing revealed a very small number of genes that are differentially expressed (Fig. 5A; Table S2 (33)), with the notable reduction of *Irs1* in female SA liver upon fasting*. Ppara* and *Cpt1a* that showed mild reduction in qPCR of female SA liver were not identified as differentially regulated genes in RNA sequencing, likely due to the smaller number of the sample size. The reduction of *Irs1* was confirmed by qPCR in female SA/SA liver but not seen in young or old male SA/SA liver (Fig. 5B-5D). Instead, *Irs2* was reduced in old SA/SA male liver but not in young male or female SA/SA liver by qPCR (Fig. 5B-5D). In the liver of WT male mice, both *Irs1* and *Irs2* were highly upregulated by fasting (Fig. 5E). Western blot confirmed IRS2 is reduced in old SA/SA male liver (Fig. 5F). However, the reduction of IRS1 was not demonstrated in SA female liver (Fig. S3K) (27). With changes in the expression of *Irs*, we compared glucose tolerance between WT and SA/SA mice. There was not a significant difference in glucose tolerance between young WT, SA het, and SA/SA male or female mice (Fig. S4A, B (27)). However, 1-year-old SA/SA male mice were mildly glucose intolerant and hyperglycemic after overnight fasting (Fig. 6A-6C). Insulin tolerance did not detect a clear difference between WT and SA male mice either at young or old age (Fig. 6D; Fig. S4C (27)). While some male mice in the SA group showed elevated fasting serum insulin, the values were not statistically significant between the 3 groups of mice (Fig. 6E). There was no significant difference in fasting glucose or serum insulin levels in female mice (Fig. S4D, E (27)). With the increase in fasting blood glucose in 1-year-old male SA/SA mice, we tested the expression of *Pck1* and *G6PC*, genes that regulates gluconeogenesis and are upregulated in WT liver after fasting (Fig. 6F). *G6PC* showed a trend of reduction in male SA/SA liver (Fig. 6G), but no changes were seen in young male and female SA/SA liver (Fig. S4F, G (27)).

### Old Male SA/SA Liver did not Show Difference in Fgf21 Expression, OXPHOS Proteins, or Inflammatory Markers

With glucose intolerance seen in old SA male mice, we probed additional genes that potentially impact glucose tolerance and reported to be regulated by PLIN5 in other tissues and cultured cells. *Fgf21* is induced in the liver by fasting in a PPARa-dependent manner (34) and its increase is reported when PLIN5 is overexpressed in the heart and skeletal muscle (35, 36). However, *Fgf21* expression was not altered in the liver of SA/SA male mice compared with WT mice upon fasting (Fig. 6H). Mitochondrial mass/function and inflammation are other pathways reported to be regulated by PLIN5 and its phosphorylation (13, 14). There was no difference in OXPHOS proteins (Fig. S5A, B (27)) or expression of inflammatory genes *Il-1b* and *Ccl2* in the liver of fasted SA/SA male mice (Fig. S5C (27)). Collectively, the loss of S155 PLIN5 phosphorylation had limited impact on glucose tolerance, mitochondrial mass, or inflammation associated with fasting in the liver except for the reduced expression of IRS2 in male mice.

## Discussion

The major finding of the current study is that S155 PLIN5 phosphorylation has a limited role for the upregulation of lipid metabolism genes that are considered to play an important role for adapting lipid metabolism upon fasting in the liver. During fasting, the liver receives NEFAs released by adipocyte lipolysis and lipid metabolism changes drastically; the liver increases LD formation, upregulates FAO, and produces ketones. These changes heavily depend on a transcription factor PPARa (18). Upon fasting, PPARa in the liver is activated by several mechanisms, including the increased expression of PPARa, the activation of coactivators such as PGC1a, and better availability of NEFAs (18). PGC1a is an especially important coactivator for upregulation of FAO genes (18). Interestingly, PLIN5 is reported to be transported to nuclei upon phosphorylation at S155, where it increases transcription of PGC1a/PPARa genes by transporting FAs to nuclei and/or increasing PGC1a activity through SIRT1 (13, 14). In addition, PLIN5 phosphorylation has been shown to increase lipolysis, a process that supports PPARa activity, providing an additional mechanism by which PLIN5 phosphorylation may support transcription of PPARa target genes (37). Indeed, the expression of a number of PGC1a/PPARa target genes is shown to be regulated by PLIN5 phosphorylation in cultured cells: *CPT1a* (hepatocytes), *PGC1a* (hepatocytes, C2C12 myoblast), *PPARa* (hepatocytes), and *PLIN5* (C2C12 myoblast) (13, 14). Thus, we assessed the contribution of PLIN5 phosphorylation in the liver upon fasting as a physiological condition in which PPARa target genes are highly upregulated and cAMP is increased (18, 19). We confirmed that phosphorylated PLIN5 is increased in the liver upon fasting. However, genes proposed to be regulated through PGC1a/PPARa during fasting in the liver showed a limited change in the fraction of genes in SA/SA female mice, with little changes in male mice. Targeted qPCR and unbiased RNA sequencing revealed little impact from the loss of S155 phosphorylation in the liver overall upon fasting, except for differential gene expression of *Irs1* in females and *Irs2* in males.

Several plausible explanations exist for the limited impact of S155 PLIN5 mutation on gene expression in the fasted liver. First, the availability of FAs as PPARa ligands might be maintained in SA/SA PLIN5 liver by mechanisms other than the transport of FAs by p-PLIN5. While a detailed kinetic study is beyond the scope of the current study, we did not observe significant changes in liver TGs. This indicates that there may not be a significant defect in lipolysis, even when PLIN5 phosphorylation is prevented in vivo. Besides PLIN5, chaperon-mediated autophagy of PLIN2 and PLIN3 could increase the access of PNPLA2 to LDs for lipolysis (38). The downregulation of *Pnpla3* during fasting (39) may increase the availability of ABHD5 to activate PNPLA2 independently from PLIN5. 17-βHSD13 also regulates PNPLA2-ABHD5 interaction as well in a PKA-dependent manner (40). Second, PGC1a/PPARa can be activated by mechanisms independent from FA availability and hepatic lipolysis. NAD^+^ and AMPK can activate PGC1a/PPARa and support the expression of target genes during fasting in the liver (41). While PNPLA2-dependent lipolysis is known to increase deacetylase activity of SIRT1 and activate PGC1a (16), SIRT1 can be directly phosphorylated by PKA as well (42). PPARa expression during fasting is upregulated by glucocorticoid that may play a dominant role in increasing transcription activity of PPARa (18). It also is plausible that other phosphorylation sites and post-translational modifications of PLIN5 compensate for the loss of S155 phosphorylation and maintain lipolysis and nuclear translocation. In the study of recombinant PLIN5 proteins, S161 and S163 were shown to be phosphorylated in addition to S155 (11). When S155A PLIN5 is overexpressed in the heart, there was an increase in S17 and S292 phosphorylation that may compensate for the loss of S155 phosphorylation (8). However, significant reduction of phosphorylated PLIN5 in the liver of SA/SA mice in Phos-Tag gel supports that S155 is the predominant site being phosphorylated in PLIN5 in vivo. In agreement, [^32^P] incorporation into recombinant PLIN5 was reduced in S155A mutant but not in S161A or S163A mutants incubated with catalytic subunit of PKA (11). Lastly, PLIN5 expression is highly upregulated in multiple tissues besides the liver upon fasting, including the heart, skeletal muscle, and pancreatic islets (5, 43). Thus, changes in lipid and glucose metabolism in other tissues might have secondary effects on the liver and eliminate the impact of the mutation in the whole-body SA/SA knock-in mice.

While PKA-dependent phosphorylation of PLIN5 is well established in cultured cell models, little is known about the extent by which PLIN5 phosphorylation changes in vivo when PKA is activated or PLIN5 expression level is increased physiologically. We utilized Phos-tag gel as a simple method to determine the proportion of phosphorylated PLIN5 in tissue protein lysates. Phos-tag gel of total liver lysate showed that the proportion of p-PLIN5/non-p-PLIN5 did not change when PLIN5 increases in the liver upon fasting or leptin deficiency, indicating that there is an increase in both phosphorylated and nonphosphorylated forms of PLIN5. In the heart, exercise significantly increased the proportion of p-PLIN5, indicating that the extent of PLIN5 phosphorylation differs dependent on tissues and context. As one proposed function of PLIN5 phosphorylation is to increase lipolysis to provide FAs for FAO, the precise regulation of FA substrate is likely more critical in the heart that primarily depends on FAO for energy production, leading to more dynamic regulation of PLIN5 phosphorylation. The difference in the proportion of phosphorylated PLIN5 may account for the extent and the direction of correlation between PLIN5 levels and PPARa target genes/mitochondrial function/FAO in different tissues. PLIN5 levels in the heart negatively correlate with mitochondria function, FAO, and PPATA/PGC1a target genes (44, 45), while a positive correlation is reported for skeletal muscle (46).

IRS2 was reduced both at mRNA and protein levels in the liver of fasted SA/SA male mice. IRS2 is considered to mediate insulin signaling primarily during fasting, while IRS1 plays a predominant role during refeeding (47). Transcriptional regulation plays an important role in increasing hepatic IRS2 expression during fasting. FOXO regulates IRS2 expression to create a feedback loop to support insulin signaling during fasting (48). Glucagon increases the CREB/CRTC2 complex and increases IRS2 expression, for which PGC1a appears to be required (48, 49). Further study is required determine whether p-PLIN5 interacts with known regulators of hepatic IRS2 expression or acts by a new mechanism during fasting in aged male mice.

Lipid and glucose homeostasis of SA/SA knock-in mice in the current study showed good agreement with mice in which SA PLIN5 was overexpressed in the liver using adenovirus-associated virus (AAV) in PLIN5 knockout mice (11). Similar to SA/SA mice, liver TG contents, TG secretion, plasma TGs, plasma FFAs, and plasma b-hydroxybutyrate were not altered in AAV-SA PLIN5 expressing PLIN5 knockout mice compared with AAV-WT PLIN5 expressing mice (11). Although lipolysis measured in liver slices ex vivo was lower in AAV-SA liver than in AAV-WT liver, the authors concluded that the overall impact of phosphorylation-resistant PLIN5 on hepatic lipid homeostasis is limited (11). Interestingly, AAV-SA PLIN5 expressing PLIN5 knockout mice showed elevated blood glucose at oral GTT compared with AAV-WT PLIN5 expressing male mice, which was associated with lower glucose-stimulated insulin secretion (11). Aged SA/SA male mice in the current study showed mild elevation in fasting glucose and elevated glucose at the intraperitoneal GTT performed after overnight fasting. As the ITT did not differ between SA/SA and WT mice, it is plausible that the glucose intolerance of SA/SA male mice is due to impaired insulin secretion. However, the serum insulin level was not reduced when fasting glucose was elevated in SA/SA mice in our study. Also, the ITT was performed after short fasting to keep blood glucose levels at time 0, which might limit the effect of SA PLIN5. Further studies are required to determine whether the reduced hepatic IRS2 level is sufficient to explain glucose intolerance or other mechanisms exist for the dysregulation of glucose homeostasis in aged SA/SA male mice.

The current study has several limitations. In LC-MS/MS analysis of semipurified LD fractions of the liver, overnight fasting increased phosphorylation of PLIN5 peptide containing S155 but not that of nonphosphorylated peptide. In comparison, both phosphorylated and nonphosphorylated PLIN5 increased after fasting in total liver lysate using a Phos-Tag gel. Fasting increases PLIN5 in the non-LD fraction, so non-p-PLIN5 may be preferentially distributed to non-LD fractions. Thus, further studies could address p-PLIN5/non-p-PLIN5 in different fractions, including the nuclear fraction. It also important to note that RNA sequencing was performed using female mice, since female mice are reported to show more changes in hepatic gene expression upon fasting (32). It is possible that RNA sequencing of SA/SA male liver may yield additional genes that are differentially regulated. Nevertheless, qPCR and Western blot did not reveal the major impact of SA/SA PLIN5 mutation on previously characterized PGC1a/PPARa targets important for FAO and mitochondrial mass in the liver. Although the impact of SA/SA knock-in is limited in fasted liver, it remains to be determined whether p-PLIN5 is required in other tissues and in a different context, especially in a tissue like the heart where the proportion of p-PLIN5 can be dynamically regulated. As sexual dimorphism exists in many genes, it requires further studies to address why hepatic *Irs1* and *Irs2* expression was differentially regulated between male and female SA/SA mice. Also, further studies are required to determine whether the subtle difference in lipid metabolism genes observed in female mice is unique to females or whether it can be detected when a larger number of male mice of a similar age are analyzed.

## Conclusion

Despite p-PLIN5's activity as a nuclear transcriptional regulator in cultured cells, PLIN5 SA/SA knock-in mice indicated that S155 phosphorylation has a limited contribution in females and is dispensable in males for the upregulation of lipid metabolism genes during the fasted state in vivo. Impairing phosphorylation also had little effect on serum lipids or liver TGs. However, SA/SA male mice showed decreased IRS2 expression in the liver, which may explain glucose intolerance in SA/SA male mice.

## Data Availability

Data will be made available on request to the corresponding author.

## Abbreviations

8-Br-cAMP: 8-bromoadenosine 3′,5′-cyclic monophosphate
AAV: adenovirus-associated virus
ABHD5: abhydrolase domain containing 5
ANOVA: analysis of variance
DMEM: Dulbecco’s modified Eagle’s medium
FA: fatty acid
FAO: fatty acid oxidation
GTT: glucose tolerance test
IP: immunoprecipitation
IRS1: insulin receptor substrate 1
IRS2: insulin receptor substrate 2
ITT: insulin tolerance test
LC-MS/MS: liquid chromatography tandem mass spectrometry
LD: lipid droplet
NEFA: nonesterified fatty acid
PGC1a: peroxisome proliferator–activated receptor-gamma coactivator
PKA: protein kinase A
PLIN1-5: perilipin 1-5
PNPLA2: adipose triglyceride lipase
PPARa: peroxisome proliferator–activated receptor alpha
RIN: RNA integrity number
SEM: standard error of mean
SIRT1: sirtuin 1
TG: triglyceride
WT: wild type
